# Histopathological effects of lactic acid bacteria enriched white soft cheese in testes of albino rats

**DOI:** 10.1038/s41598-025-26073-2

**Published:** 2025-11-28

**Authors:** Ruwaida Elhanbaly, Khaled H. Salman, Fatma M. Abdel-maksoud, Fatma Abo Zakaib Ali

**Affiliations:** 1 Department of Anatomy and Embryology, Faculty of Veterinary Medicine, Assiut University, Assiut, 71515 Egypt; 2 Department of Dairy Science, Faculty of Agriculture, Al-Azhar University, Assiut, 71524 Egypt; 3 Department of Pathology and Clinical Pathology, Faculty of Veterinary Medicine, Sohag University, Sohag, 82524 Egypt

**Keywords:** White brined cheese, *L. helveticus*, *L. rhamnosus*, *S. thermophilius*, Testes, Immunohistochemical, Biological techniques, Cell biology

## Abstract

Soft cheeses provide an ideal matrix for bacterial growth, serving as a medium for inoculating starter microorganisms like Lactobacillus. This study aimed to evaluate the biochemical, histopathological, and immunohistochemical effects of white soft cheese inoculated with *Lactobacillus helveticus*,* Lactobacillus rhamnosus*,* and Streptococcus thermophilus S3855* on male reproductive health in albino rats. Forty male Albino rats, divided into five isolated groups, and fed white soft cheese prepared with different bacterial strains or non-inoculated cheese (control) for 28 days. The prepared cheese was pickled for four weeks at refrigerator temperature and analyzed for chemical and microbiological properties at 7, 14, 21, and 28 days. Total solids decreased over time, while protein-to-dry matter ratios increased across all treatments. Biochemically, all treated groups showed reduced serum total cholesterol, with the most significant triglyceride reduction in group V. Histopathological analysis revealed notable structural damage in testicular tissue, including degeneration of seminiferous tubules, vascular abnormalities, and a marked decrease in seminiferous tubule diameter and spermatogenic cell counts. Johnsen-like scores were significantly lower, particularly in groups III, IV, and V, indicating impaired spermatogenesis compared with the normal control. Immunohistochemically, strong Bax expression was observed in all treated groups, indicating enhanced apoptotic activity. Additionally, wide spread Plexin-B1 expression was also detected in all treated groups. These findings indicate that probiotic-enriched soft cheese may negatively affect male reproductive health, possibly through apoptosis-mediated pathways.

## Introduction

North Africa, Eastern Europe, the Middle East, and the Balkans are the places where brined white cheese is most popular. They include a great variety of variant cheeses, such as Domiati cheese (in Egypt), Feta (in Greece), Telemea (in Romania, Greece), Beyazpeynir (in Turkey), Akawi (in lebanon, Syria), and Halloumi (in Cyprus). Cheeses are manufactured by varied processing processes; therefore, they have disparities in their chemical, physical, textural, and sensory features^1,2^. Soft cheeses have an excellent matrix for the development of various types of bacteria, and they provide an excellent medium for transferring inoculated starter microorganisms and transforming the product into a useful food. In fact, cheese contains a good amount of fat, calcium, moisture, and water activity in addition to a solid protein structure and a nearly neutral pH that promotes the growth of microorganisms and ensures their viability for the duration of the dairy product’s storage life^3,4^.

The most widely used bacteria are specifically those belonging to the Lactobacillus species, which are thought to be the most appropriate for use in food formulations due to their natural presence in a wide range of fermented foods^5,6^. Starter bacterial integration, particularly concerning Lactobacillus, has been extensively researched in relation to various cheese varieties^3^.

The liver and kidneys of the male albino rats (Albino *Rattus norvegicus*) were significantly affected by feeding on cultured white soft cheese. Several pathological changes were present in the liver and kidneys of all experimental groups away from the control rats^7^. As the testes are the main constituent of the male reproductive system, they as crucial organs that determine the function of the male reproductive system. The testes not only play an important role in male reproductive endocrine functions but are also considered significant sites of sperm development and regulation^8^. The function of the testes is principally attained through the collaboration of Sertoli, Leydig, and germ cells. Sertoli cells supply structural support for the secure progression of germ cells through multiple rounds of mitosis and meiosis^9^. Leydig cells principally maintain male secondary sexual characteristics through the manufacturing and secretion of the testosterone hormone^10^. The existence of testosterone is pivotal for sustaining the development of sperm, and its suppression perhaps leads to disturbance in the spermatogenesis process^11^. The blood-testicular barrier provides a microenvironment convenient for spermatogenesis, offers immune protection, and imparts polarity to epithelial germ cells^12^.

The designed study aimed to investigate the effect of cultured white soft cheese on the histopathological and immunohistochemical alterations in the testes of rats inoculated with *L. helveticus*, *L. rhamnosus*, and *S. thermophilus* S_3855_, and monitor the changes in the chemical composition of cheese during the pickling period.

## Materials and methods

The recent study was conducted following Egyptian guidelines and University regulations for animal care. All experimental and euthanasia procedures were performed in agreement with a protocol approved by the Ethical Committee of the Faculty of Veterinary Medicine, Assiut University, Assiut, Egypt (Approval No. 06/2023/0135), following the OIE standards for the use of animals in research. The study also complies with the ARRIVE guidelines (https://arriveguidelines.org). Albino rats (Albino Rattus norvegicus) weighing 150–250 g were obtained from the farm of the National Organization for Drug Control and Research, Giza, Egypt. Rats were housed in rooms with a temperature of 25 °C and 55 °C.% R.H. were housed inside aluminum cages with a mesh bottom for 28 days and fed on a basal diet (maize starch 70%, crude protein 12%, corn oil 8%, fiber 5%, salt mixture 4%, and vitamin mixture 1%) for one week.

### Cheese manufacturing

Fresh cow’s milk was obtained from the farm of the Animal Production Department, Faculty of Agriculture, Al-Azhar University (Branch of Assiut). Microbial rennet powder (Fromase R 2200) was obtained from DSM (France). Salt: Sodium chloride was obtained from the Company of El-Nasr for salt (Alexandria, Egypt).

Starter strains: *Lactobacillus rhamnosus* was obtained from the Dairy Department, Faculty of Agriculture, Al-Azhar University. *Lactobacillus helveticus* (ATCC15009) was obtained from the Cairo Microbiological Resource Center (MIRCEN), Faculty of Agriculture, Ain Shams University. *Streptococcus thermophilus* S_3855_ encapsulated strain was obtained from the Department of Dairy Science, Faculty of Agriculture, Minia University.

White soft cheese was manufactured using the traditional method of creating Domiati cheese by IDF^13^(International Dairy Federation), with some modification according to Salman et al^7^.. For getting four types of cheese, one cheese of non-starter (Control Cheese) and three cheeses made with 0.75% (w/w) of each *Lb. helveticus* bacteria, *Lb. rhamnosus* bacteria and *S. thermophilus* S_3855_ bacteria.

### Animal study

Forty male Albino rats were divided into five groups randomly and evenly (eight rats each). After the 7-day acclimatization period, each group had one of the following diets every day for 28 days: Group1 (control group) was fed a basal diet, while groups 2 to 5 were fed a 70% basal diet in addition to 30% of control cheese, cheese contains *L. helveticus*, cheese contains *L. rhamnosus* and cheese contains *S. thermophilus* S_3855_ respectively.

### Chemical analysis

Total solids and total nitrogen contents were determined according to IDF standards^13,14^, while fat content was measured following AOAC^15^.

At the end of the experiment, 2 ml of blood was collected from each rat via the retrobulbar venous plexus using EDTA-coated glass tubes^16^. The samples were then immediately centrifuged for 15 min at 4000 rpm. Serum total cholesterol and triglyceride levels were then analyzed^17^, with triglyceride determination also following the procedure of Fossati and Principe^18^.

## Histopathological studies

### Specimen processing and staining

At the end of the respective experimental periods, the animals from each group were weighed and then anesthetized via intraperitoneal injection of equithensin (1 mL/kg). After the loss of all reflexes, the animals were trans-cardiac perfused with warm saline followed by Trump’s fixative (3.7% formaldehyde plus 1% glutaraldehyde in saline buffer)^19^. Following perfusion, the testes were collected and immersed in 4% paraformaldehyde (catalog no. 19200; lot no. 090820; Electron Microscopy Sciences, JEOL, Tokyo, Japan) for further processing. Finally, the deeply anesthetized animals were humanely euthanized by cervical dislocation, performed by a proficient person in accordance with the AVMA guidelines for the euthanasia of animals^20^.

The samples were dissected and then immediately fixed in 10% PBS-formalin for 24 h, after which they were dehydrated in a graded concentration of alcohol, cleared in xylene, and finally embedded in paraffin. To prevent repetitive assessment of the same structures, sections were obtained at regular intervals of approximately 50 μm throughout the tissue block. The sections were then stained with hematoxylin and eosin (H&E)^21–23^.. The sections were examined for histopathological changes using a light microscope, OLYMPUS CX43. Photographs of the sections were taken using an OLYMPUS DP72 camera adapted to the microscope.

### Evaluation of spermatogenesis

Spermatogenesis was valued using the Johnsen-like score^24^, an adaptation of the original Johnsen scoring system commonly used for assessing human spermatogenesis—for application in rats, as described by Oliveira Filho et al.^25^. In each biopsy, 50–100 cross-sections of seminiferous tubules were examined following the methods of Filipiak et al^26^. and Soliman et al.^22^, as presented in Table 1.

**Table 1:** Adaptation of the Johnsen-like score^24^ for the evaluation of spermatogenesis in rat.

Score	Evaluation of spermatogenesis	Score	Evaluation of spermatogenesis
10	Complete spermatogenesis with mature sperm cells	9	Some sperm cells, with a disorganized epithelium
8	Presence of a few sperm cells (< 5 to 10)	7	No sperm cells, presence of spermatids
6	No sperm cells, few spermatids (< 5 to 10)	5	No sperm cells or spermatids, presence of spermatocytes
4	No sperm cells or spermatids, few spermatocytes (< 5)	3	Only spermatogonia present
2	Sertoli cells only	1	No cells were visualized in the tubular cross-section

Microscopic vascular changes were scored according to severity in the examined tissue: 0 = no lesions; 1 = minimal (1 to 10% of the tissue section affected); 2 = mild (11 to 25%); 3 = moderate, (26 to 45%); 4 = severe (> 45%)^27,28^.

### Morphometric measurement

The morphometric studies were carried out on the H & E-stained sections by using ImageJ versus 1.48 software (NIH). It was used for measuring the area of seminiferous tubules/µm^2^, height of spermatogenic cells (SGs), and count of both Sertoli (SCs) and SGs. Calculation of spermatogenic index (SI); SG count/SC count was done. Five non-overlapping sections from each paraffin block were taken within these sections, seminiferous tubules were selected randomly but under consistent conditions, excepting distorted or overlapping profiles. Tubular area and germinal epithelium height were measured, while Sertoli and spermatogenic cell counts were performed at high-power fields (×400) within a standardized area of 293.4288 μm² using ImageJ software. On average, 10–25 Sertoli cells and 20–100 spermatogenic cells were counted per animal^22,29^.. Morphometry was carried out at the Image Analysis Unit, Department of Pathology and Clinical Pathology, Faculty of Veterinary Medicine, Sohag University. All morphometric and histological evaluations were carried out in a blinded manner to ensure reproducibility and minimize observer bias.

### Immunohistochemistry studies

Small specimens from the body and tail regions were fixed in 4% paraformaldehyde in 0.1 ML phosphate-buffered saline (PBS, pH 7.4) overnight at 4 °C. After that, the paraffin sections were deparaffinized in xylene, hydrated in a descending concentration of alcohol, and washed with 0.1 ML PBS (3 × 10 min). Then making an antigen retrieval to reduce the masking of antigen epitopes, was performed using 0.1 ML sodium citrate buffer solution (pH 6) for 10 min in a microwave (600 W). The sections were then cooled to room temperature for 30 min and washed with PBS (pH 7.4), for 15 min. After blocking the endogenous peroxidase activity with 3% H_2_O_2_ in H_2_O for 30 min at the room temperature (RT), then sections were washed with PBS (3 × 5 min). Afterwards, the sections were blocked with 10% normal goat serum (NGS) + 0.2% Triton-X100/PBS for 2 h at RT. Later, sections were incubated overnight at 4 °C with the following antibodies: Bax (1:100, Elabscience, catalog no. E-AB-13814) and Plexin-B1 (1:500 Abcam catalog no. ab39717). Sections were incubated with biotinylated IgG goat anti-rabbit secondary antibody (Dako, Hamburg, Germany) diluted at 1:250 for 2 h at RT, followed by incubation with Vecta stain ABC (Avidin-Biotin complex) reagent for 45 min in a humid chamber at room temperature. Visualization of the reaction was carried out with DAB for 5–10 min. The sections were counterstained with Harris hematoxylin. The staining was examined by Leitz Dialux 20 Microscope. The photos were captured by a digital camera (Canon PowerShot A95)^30,31^. The staining quantification was carried out using an NIH ImageJ version 1.53i software (National Institutes for Health, NIH, Bethesda, MD).

### Statistical analysis

In order to determine normality, the Shapiro-Wilk test was conducted before analysis, and the findings were not statistically significant. Data was analyzed using Analysis of Variance (one-way ANOVA) in the Statistical Analysis System (SAS). Mean differences were assessed with the Tukey–Kramer HSD test and presented as means ± standard error (SE). For histomorphometric assessments, data were expressed as means ± standard deviations (SD) using one-way ANOVA (non-parametric) based on the Kruskal-Wallis test followed by Dunn’s multiple comparisons post hoc test. Significant differences compared to the control group and among treatment groups were indicated by different asterisks, (^*^p ≤ 0.05, ^**^p ≤ 0.01, ^***^p ≤ 0.001).

## Results

### Effect of lactic acid bacteria on the chemical composition of white soft cheese

Data in Table 2 showed that the addition of lactic acid bacteria had a significant effect (*p* ≤ 0.05) on total solids, protein/DM, and fat during storage of cheese in the refrigerator up to 28 days. The highest values of total solids were obtained in *L. rhamnosus* cheese when fresh (36.0%), followed by *L. helveticus* cheese when fresh (36.0%), while the lowest values were obtained in *L. helveticus* cheese (27.24%) at the end of pickling time (28 days). Regarding Protein/DM content, the highest values were obtained in *S. thermophilus* S3855 cheese (ranging from 40.65% to 46.96%), while the lowest values were obtained in control cheese, which ranged from 33.27% to 35.68%. The results indicate that the treatments containing lactic acid bacteria had higher values in protein/DM than those found in control cheese. Regarding fat content, the highest value in fat content was obtained in *L. rhamnosus* cheese (11.06%) when fresh, followed by S. thermophilus S3855 cheese (10.80%) when fresh, while the lowest value was found in S. thermophilus S3855 cheese (8.26%) at the end of pickling time. The data reveal that the fat content of white soft cheese dropped and changed significantly (*p* ≤ 0.05) while kept in the refrigerator for 28 days.

**Table 2:** Chemical composition of white soft cheese held at refrigerator temperature for up to 28 days.

Treatments	Mean (%) ± SD			
	Storage periods	Total solids	Protein/DM	Fat
**Control Cheese**	Fresh	35.67 ± 0.95^abc^	33.27 ± 1.23^g^	10.40 ± 0.00^bc^
	7 days	34.90 ± 0.45^abcde^	33.40 ± 1.38^g^	10.30 ± 0.00^bcd^
	14 days	31.57 ± 0.31^efgh^	33.89 ± 7.02^fg^	9.86 ± 0.05^cdef^
	21 days	31.43 ± 0.95^efghi^	34.58 ± 4.63^efg^	9.76 ± 0.05^def^
	28 days	30.87 ± 0.20^fghi^	35.68 ± 3.63^defg^	9.76 ± 0.15^def^
**Cheese made with** ***L. helveticus*bacteria**	Fresh	36.00 ± 3.99^ab^	38.41 ± 1.75^bcdefg^	10.33 ± 0.37^bcd^
	7 days	35.00 ± 0.40^abcde^	42.30 ± 0.65^abcd^	10.00 ± 0.10^cdef^
	14 days	32.03 ± 0.76^defg^	43.48 ± 1.23^abc^	9.66 ± 0.11^ef^
	21 days	27.91 ± 0.74^ij^	43.75 ± 0.86^abc^	8.76 ± 0.11^h^
	28 days	27.24 ± 0.04^j^	44.88 ± 1.05^ab^	8.76 ± 0.05^h^
**Cheese made with** ***L. rhamnosus*** **bacteria**	Fresh	38.31 ± 0.35^a^	36.62 ± 0.14^cdefg^	11.06 ± 0.05^a^
	7 days	33.55 ± 1.16^bcdef^	41.04 ± 0.75^abcdef^	10.06 ± 0.05^cde^
	14 days	32.32 ± 0.39^cdefg^	41.16 ± 1.29^abcde^	9.86 ± 0.05^cdef^
	21 days	31.82 ± 0.74^defgh^	42.07 ± 0.29^abcd^	9.83 ± 0.05^cdef^
	28 days	30.83 ± 0.05^fghi^	43.81 ± 0.69^ab^	9.73 ± 0.05^def^
**Cheese made with** ***S. thermophilus*** **S**_**3855**_ **bacteria**	Fresh	35.20 ± 1.40^abcd^	40.65 ± 0.45^abcdef^	10.80 ± 0.43^ab^
	7 days	32.91 ± 1.38^bcdefg^	41.19 ± 1.46^abcde^	10.10 ± 0.50^cde^
	14 days	30.75 ± 0.70^fghij^	45.48 ± 2.29^ab^	9.40 ± 0.26^fg^
	21 days	29.53 ± 0.85^ghij^	46.41 ± 1.44^a^	8.80 ± 0.17^gh^
	28 days	28.25 ± 0.23^hij^	46.96 ± 1.13^a^	8.26 ± 0.05^h^
**P-value**		< 0.001	< 0.001	< 0.001
**df**		19	19	19
**F-value**		19.20	11.10	38.30
**(χ2)**		55.0	47.40	-
**C.V.**		3.58	5.75	2.01

Means, in the same column, followed by the same letter are not significantly different using the Tukey–Kramer HSD test at *P* ≤ 0.05.

### Effect of cheese enriched with lactic acid bacteria on levels of serum total cholesterol and triglycerides in rats

Data in Table 3 indicates that there is a clear variation and significant difference (*P* ≤ 0.05) in TC values before and after feeding. The serum levels of the final TC for all treated groups were significantly lower than those of the initial samples. Moreover, feeding the rats with cultured cheese appears to cause various decreases in TC values. Whereas the group fed cheese containing *L. rhamnosus* had a higher reduction in TC value (from 116.01 to 79.20 mg/dL), followed by the group fed cheese containing *L. helveticus*, while the group fed cheese containing *S. thermophilus S*_*3855*_ had the lowest reduction in TC level.

The effects of the experimental diets on the levels of serum TG showed a significant difference in triglycerides among the five groups at the beginning and at the end of feeding. The treatment of Group **GV** (fed on *S. thermophilus* S_3855_ cheese) has the highest reduction value of TG, from 78.26 mg/dL to 55.57 mg/dL, while the treatment of Group **GI** (fed on a basal diet) has the lowest reducing value at the end of the feeding period (Table 3).

**Table 3:** Levels of serum total cholesterol and triglycerides in albino rats before and after fed on white soft cheese.

Rats Groups	Mean ± SD			
	Total Cholesterol (mg/dL)		Triglycerides (mg/dL)	
	Before feeding	After feeding	Before feeding	After feeding
**GI**	101.79 ± 9.86^ab^	86.59 ± 1.94^a^	58.42 ± 1.32^c^	55.57 ± 2.73^a^
**GII**	94.77 ± 5.92^bc^	67.76 ± 6.28^b^	60.80 ± 2.03^c^	55.07 ± 1.09^a^
**GIII**	94.43 ± 4.41^bc^	59.70 ± 2.25^b^	67.51 ± 2.45^b^	54.90 ± 3.12^a^
**GIV**	116.01 ± 3.32^a^	79.20 ± 3.58^a^	76.17 ± 3.45^a^	54.03 ± 2.11^a^
**GV**	84.18 ± 1.15^c^	81.59 ± 2.70^a^	78.26 ± 1.01^a^	55.57 ± 4.72^a^
**P-value**	< 0.001	< 0.001	< 0.001	< 0.001
**df**	4	4	4	4
**F-value**	12.60	26.30	47.50	0.13
**(χ2)**	6.11	3.19	2.88	3.22
**C.V.**	5.83	4.94	3.27	5.46

Means, in the same column, followed by the same letter, are not significantly different using the Tukey–Kramer HSD test at *P* ≤ 0.05.

### Histopathological assessment

**Fig. 1:**
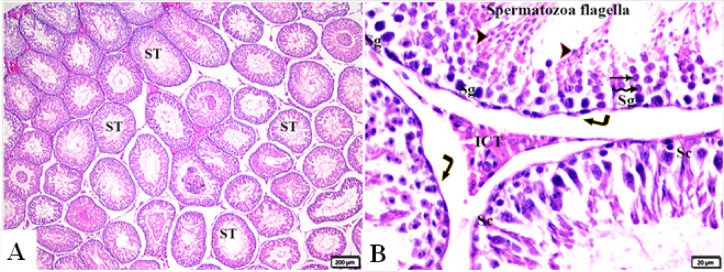
Photomicrograph of rat testicular tissue from normal control group I showing: **(A)**: Normal histological seminiferous tubules (ST). **(B)**: Normal structured seminiferous tubule, lined by germ cells in order from basal lamina to center as spermatogonia (Sg), primary spermatocytes (Zigzag arrows), secondary spermatocytes (thin arrows) and spermatids (arrowheads). The Sertoli cells (Sc) were found throughout the entire thickness of the germinal epithelium from the basal lamina to the spermatids. Spermatozoa flagella fill the tubule lumen. Normal myoid cell rest on basement membrane (elbow arrows). Hematoxylin and Eosin staining. The scale bar size is A = 200 μm, B = 20 μm.

The histological pattern of the testis of the control group showed the normal histological structure of seminiferous tubules (ST), including the shape and arrangement of their cellular components (Fig. 1A, B). Spermatogonia (Sg) and Sertoli cells (SCs) rest on intact basement membranes. Large primary spermatocytes (PS) with characteristic large, rounded nuclei, round spermatids, elongated spermatids, and late-stage sperms (S) attached to the apices of SCs were seen (Johnsen-like score 10) (Fig. 1C).

**Fig. 2:**
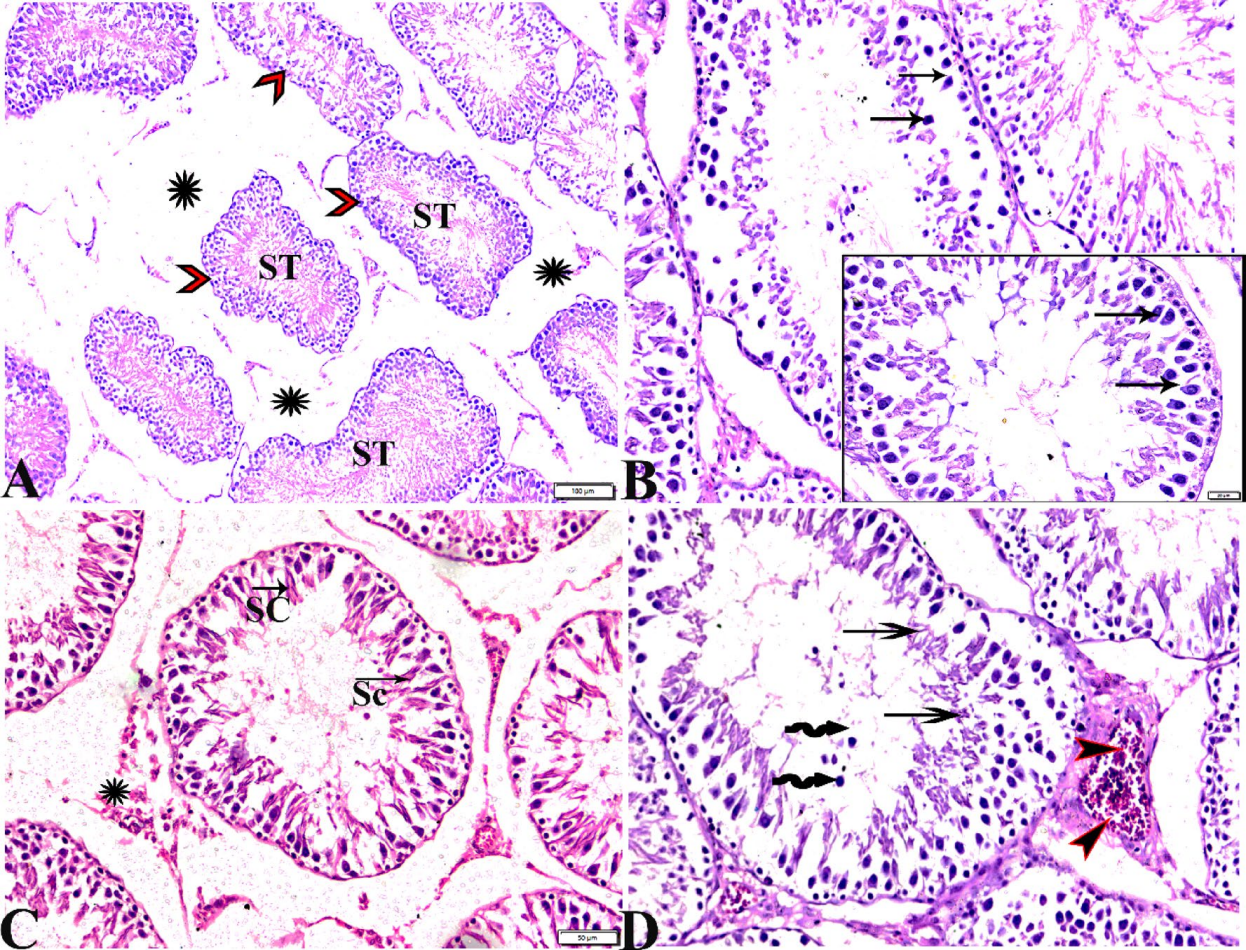
Photomicrograph of rat testicular tissue from group II (treated with basal diet 70% + control cheese 30%) showed: **(A)**: wrinkled and buckled basement membrane of seminiferous tubules (red arrowheads), tubular deformation, degeneration and shrunken seminiferous tubules (ST), sever edematous fluid in between seminiferous tubules (Star). **(B)**: Loss of germ-cell attachment, in most tubules the majority of the germinal epithelium was lined by spermatogonia (arrows) and Sertoli cells (SC) (C, arrows). **(C)**: Reduced Leydig cell population (Star). **(D)**: Necrotic spermatogenic epithelium (arrows), germinal cells sloughed into tubular lumen (zigzag arrows), sever congested vessel (arrowheads). Hematoxylin and Eosin staining. The bar size was indicated under pictures.

The microscopic examination of testicular sections from group II (treated with basal diet 70% + control cheese 30%) revealed a mosaic of different seminiferous tubules with varying tubular deformation, degeneration, and shrunken seminiferous tubules (ST) (Fig. 2A, B). Although reduced spermatogenesis, tubules with maturation were arrested up to the level of the spermatocytes (Fig. 2C, D). The ST showed numerous morphological abnormalities such as; severe edematous fluid in between seminiferous tubules, vascular congestion, and separation of basal and luminal cellular compartments in some ST with loss of germ-cell attachment. The majority of tubules showed many exfoliated cells in the tubular lumen and the germinal epithelium was lined by spermatogonia and Sertoli cells (SC) only. Leydig cell population was reduced (Fig. 2C, D).

**Fig. 3:**
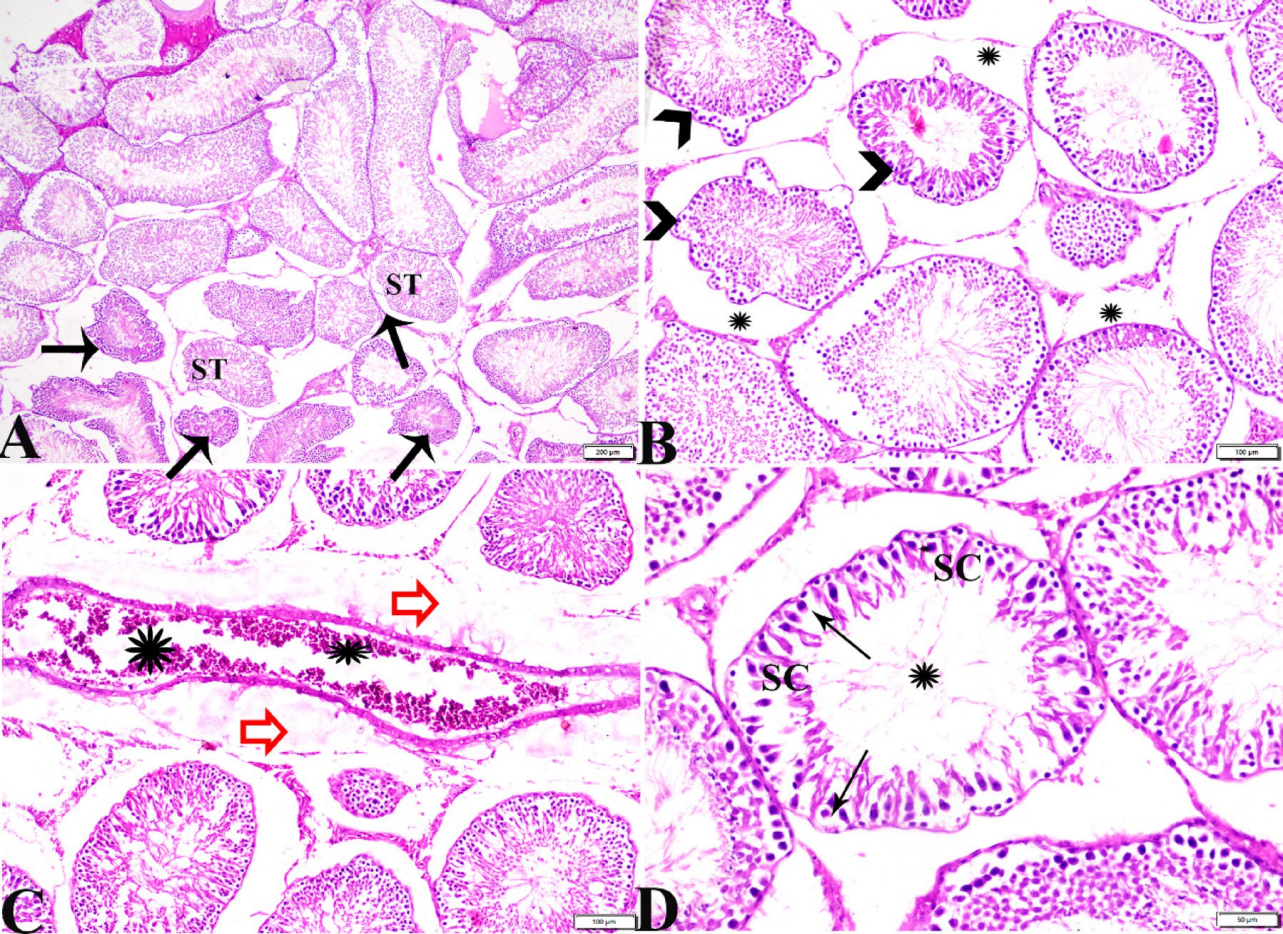
Photomicrograph of rat testicular tissue from group III (treated with basal diet 70% + cheese contains *L. helveticus*30%) showing: **(A)**: Atrophic seminiferous tubules (ST) (arrows). **(B)**: buckled basement membrane of degenerated seminiferous tubules (arrowheads) with interstitial edema in-between (Stars). **(C)**: sever dilatation and congestion in blood vessel (stars) with marked perivascular edema (arrows). **(D)**: Seminiferous tubule showing disruption of intercellular junctions between germ cells and lined only by spermatogonia (arrows) and Sertoli cells (SC) with vacuolated cytoplasm. Note: The tubular lumen was clear from any sperm (star). Hematoxylin and Eosin staining. The bar size was indicated under pictures.

Histopathological examination of testicular tissue from group III (treated with basal diet 70% + cheese containing *L. helveticus* 30%) showed a generalized picture of degenerated seminiferous tubular associated with irregular shrink basement membrane. Interstitial edema in-between ST (Fig. 3A, B). Marked vascular dilatation and congestion with perivascular edema (Fig. 3C). Disrupted intercellular junctions between germ cells and spermatogenic arrest show only spermatogonia and vacuolated Sertoli cells in most tubules with no sperms and few elongated spermatids in the lumen of ST (< 5 to 10, Johnsen-like score 6) (Fig. 3D).

**Fig. 4:**
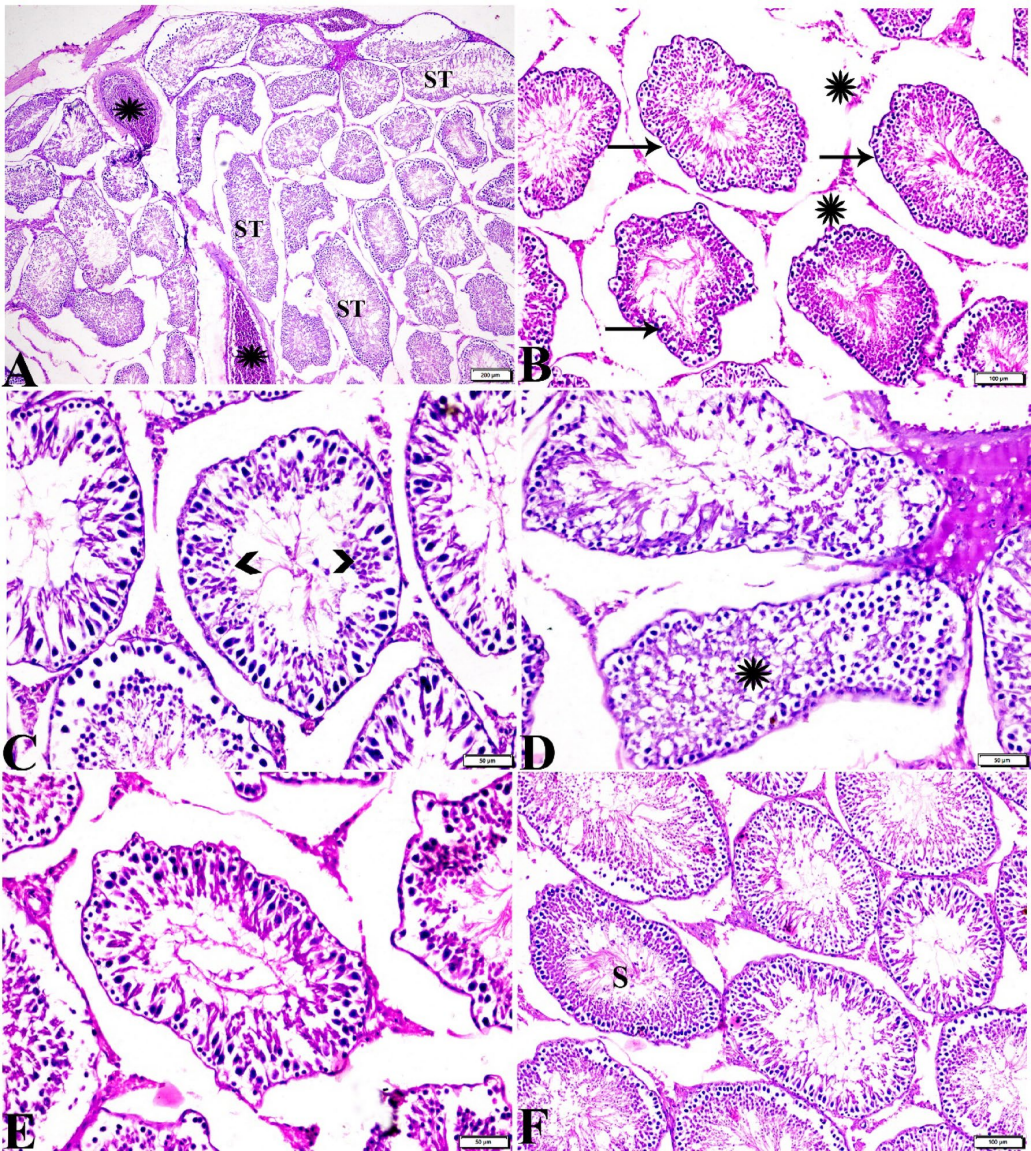
Photomicrograph of rat testicular tissue from group IV (basal diet 70% + cheese contains *L. rhamnosus*30%) showing: **(A)**: Atrophy in seminiferous tubules (ST), sever vascular congestion (stars). **(B)**: wrinkled basement membrane (arrows), interstitial edema (stars). **(C)**: Necrotic spermatogenic epithelium with sloughed spermatogonia into tubular lumen (arrowheads). **(D)**: Tubular deformation and disorganization of spermatogenic epithelium (star). **(E)**: Seminiferous tubule showing more or less normal spermatogenic epithelium. **(F)**: some tubules have mature sperms (S) inside its lumen. Hematoxylin and Eosin staining. The bar size was indicated under pictures.

Testicular tissue sections from group IV (basal diet 70% + cheese containing *L. rhamnosus* 30%) demonstrated atrophied seminiferous tubules (ST) with wrinkled basement membrane, separated by wide area of interstitial edema (Fig. 4A-C). Necrotic spermatogenic epithelium with presence of sloughed spermatogonia germ cells into tubular lumen (Fig. 4C). Some ST showed varies morphological deformity and disorganization of spermatogenic epithelial cells, although some seminiferous tubule showed more or less normal spermatogenic epithelium with the Presence of few sperm cells (< 5 to 10) inside its lumen (Johnsen-like score 7,8) (Fig. 4D-F).

**Fig. 5:**
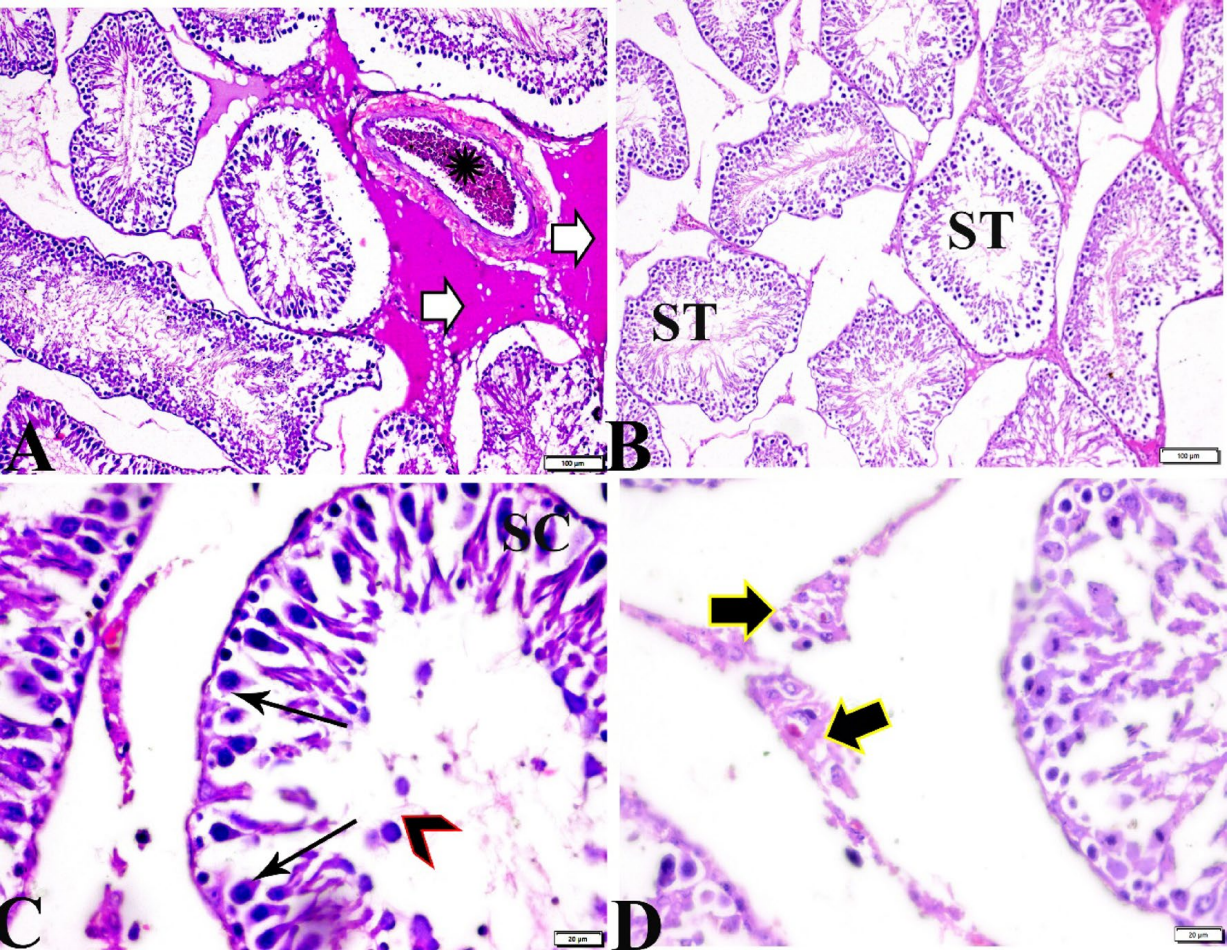
Photomicrograph of rat testicular tissue from group V (basal diet 70% + cheese contains *S. thermophilus* S_3855_30%) showing: **(A)**: sever vascular congestion (star), perivascular hemorrhage (arrows). **(B)**: Atrophied seminiferous tubules (ST) with irregular basement membrane. **(C)**: Seminiferous tubule lined only by spermatogonia (arrows) and Sertoli cells (SC) with vacuolated cytoplasm, sloughed germinal cells into tubular lumen (arrowheads). **(D)**: Reduced Leydig cell population with alterations (arrows). Hematoxylin and Eosin staining. The bar size was indicated under pictures.

Histopathological examination of testicular tissue sections from group V (basal diet 70% + cheese containing *S. thermophilus* S_3855_ 30%) showed variable sizes of atrophied seminiferous tubules (ST) with irregular basement membrane lining. Seminiferous tubule lined only by spermatogonia and vacuolated Sertoli cells (SC) (Johnsen-like score 4, 5). Germ cells lost their intercellular junctions, and some germinal cells were sloughed into tubular lumen (Fig. 5B, C). Leydig cell showed some morphological alterations and a reduction in its density (Fig. 5D). Severe vascular congestion and perivascular hemorrhage were also noticed in this group (Fig. 5A).

### Quantitative and semiquantitative histomorphometry studies

#### Johnsen-like scores

**Fig. 6:**
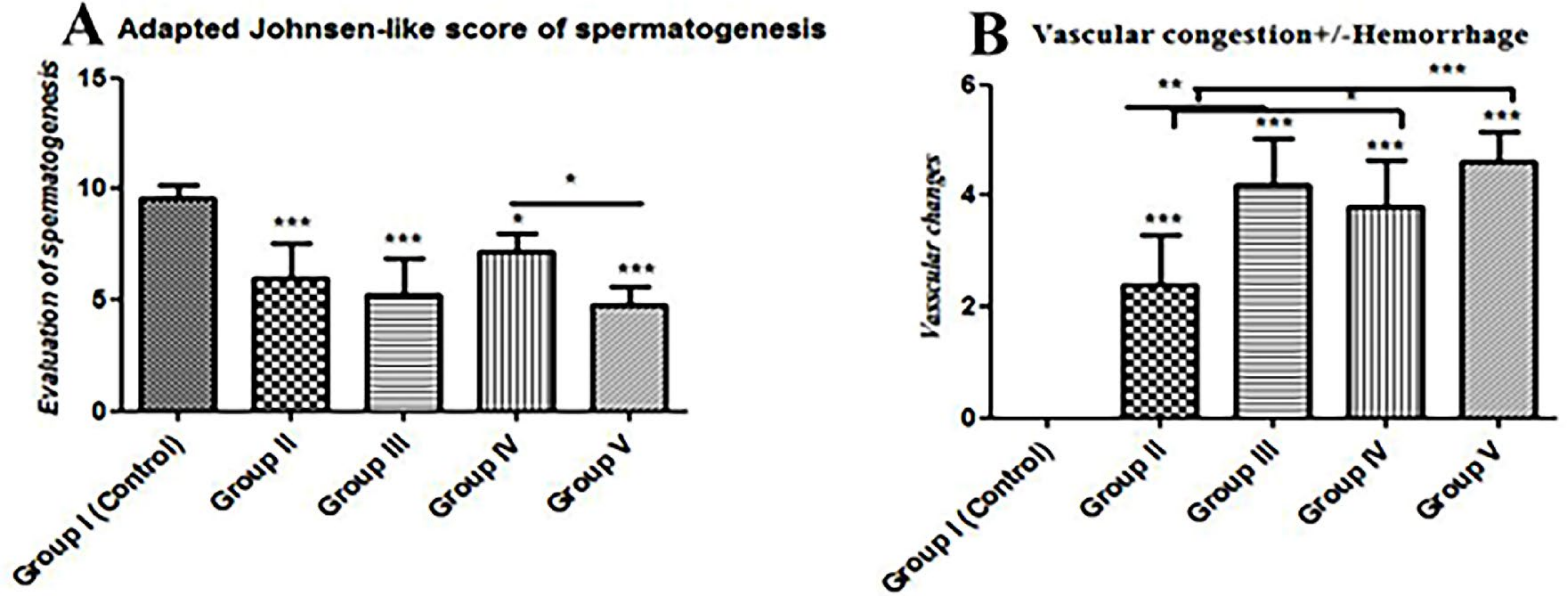
Histomorphometry graph showing semiquantitative measurements of testicular tissue sections among the experimental groups: **(A)**: Adapted Johnsen-like scores of spermatogenesis in all studied groups, **(B)**: Vascular congestion with or without perivascular hemorrhage. Data are expressed as means ± standard deviations. Significant differences vs. the control group are marked by different asterisks through Kruskal-Wallis test with Dunn’s Multiple Comparison post hoc test: * *p* ≤ 0.05, ** *p* ≤ 0.01, *** *p* ≤ 0.001).

Regarding the mean Johnsen-like scores of the experimental groups, they were high significantly decreased (*p* ≤ 0.05) in comparison to the control group. Moreover,

the mean score of group IV (basal diet 70% + cheese contains *L. rhamnosus* 30%) treated rats were significantly (*p* ≤ 0.05) lower than that of group V (basal diet 70% + cheese contains *S. thermophilus* S3855 30%) (Fig. 6A).

### Vascular changes

Although all treatments induced a significant (*p* ≤ 0.05) increase in the severity of vascular changes that present in dilated blood vessels engorged with blood associated with perivascular hemorrhage in some cases as compared to control animals, the effect was much more pronounced significantly (*p* ≤ 0.05) in Groups III, IV, and V compared with Group II (Fig. 6B).

### Diameter of seminiferous tubules

**Fig. 7:**
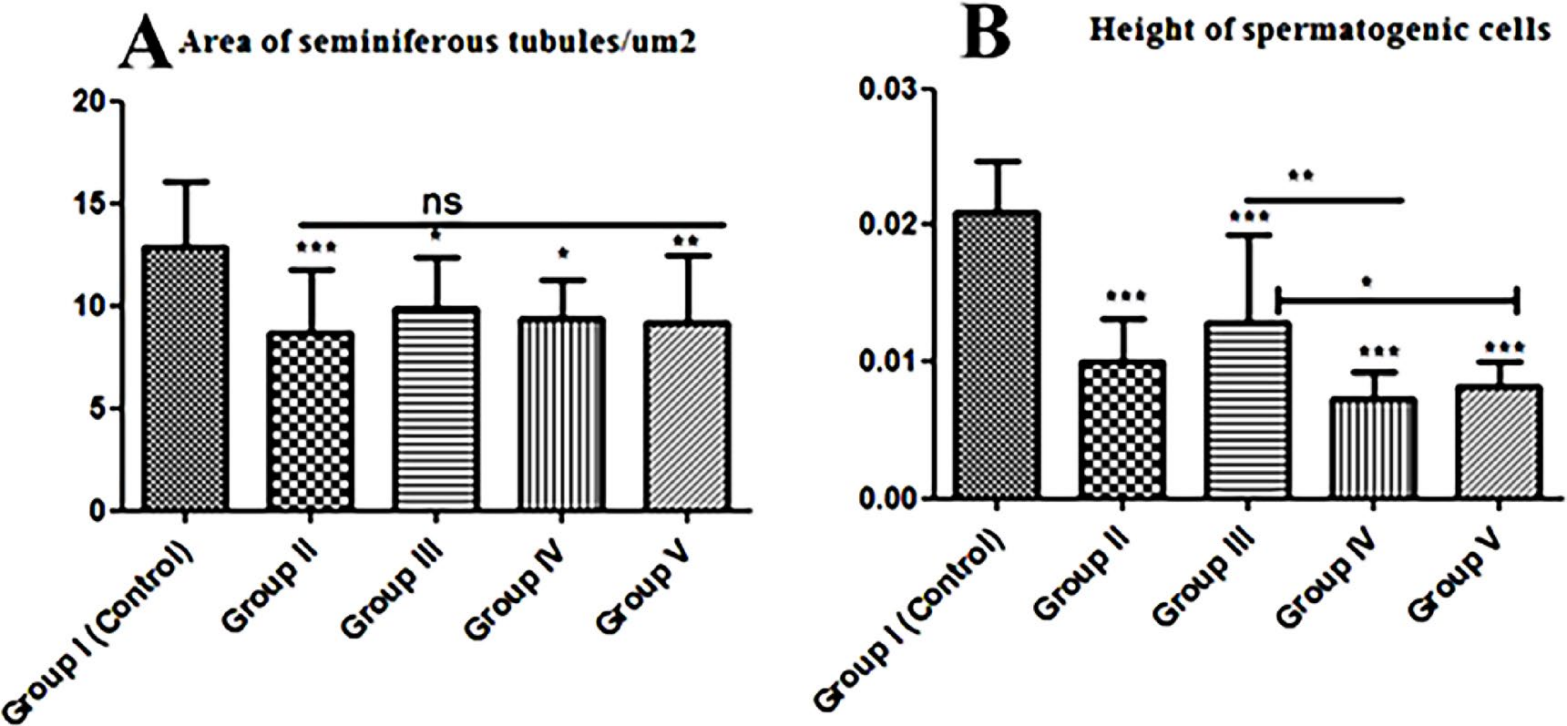
Histomorphometry graph showing quantitative measurements of testicular tissue sections among the experimental groups: **(A)**: Area of seminiferous tubules/µm2 in all studied groups, **(B)**: Height of spermatogenic cells (SGs) of seminiferous tubules. Data are expressed as means ± standard deviations. Significant differences vs. the control group are marked by different asterisks through Kruskal-Wallis test with Dunn’s Multiple Comparison post hoc test: * *p* ≤ 0.05, ** *p* ≤ 0.01, *** *p* ≤ 0.001).

Control cheese, *L. helveticus*, *L. rhamnosus*, *and S. thermophilus* S_3855_ bacteria added to the basal diet induced a significant decrease in the diameter of seminiferous tubules in comparison to the control group (Fig. 7A). On the other hand, the mean epithelial height of seminiferous tubules significantly (*p* ≤ 0.05) reduced in comparison with the control untreated group. Between treated groups, there are high a significant (*p* ≤ 0.05) reduction in the spermatogenic epithelial height in Group IV rats compared with Group III rats, and a slight significant (*p* ≤ 0.05) reduction in Group V than in Group III (Fig. 7B).

### Spermatogenic cell (SGs) count, Sertoli cell (SC) count

**Fig. 8:**
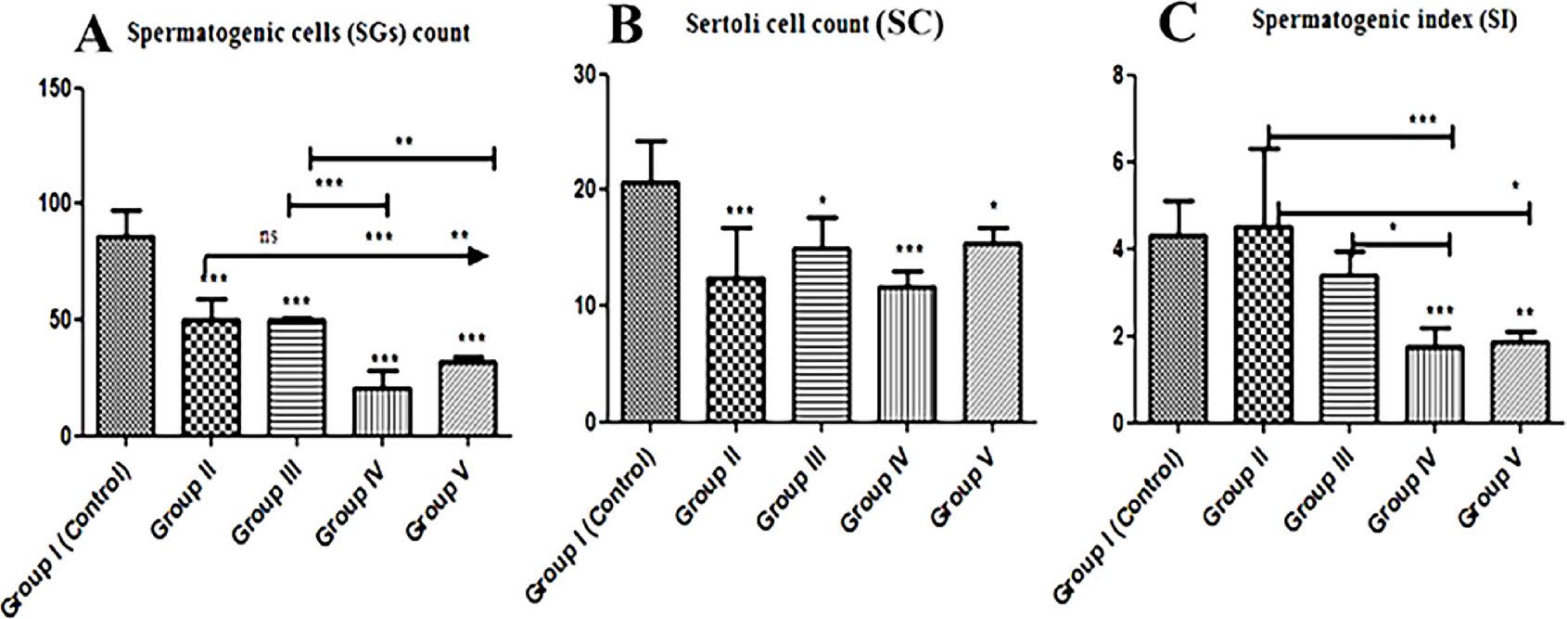
Histomorphometry graph showing quantitative measurements of testicular tissue sections among the experimental groups: **(A)**: Spermatogenic cell (SGs) count **(B)**: Sertoli cell (SC) count. **(C)**: spermatogenic index (SI); SG count/SC count. Data are expressed as means ± standard deviations. Significant differences vs. the control group are marked by different asterisks through Kruskal-Wallis test with Dunn’s Multiple Comparison post hoc test: * *p* ≤ 0.05, ** *p* ≤ 0.01, *** *p* ≤ 0.001).

The mean count of spermatogenic cells (SGs) was significantly (*p* ≤ 0.05) decreased in all groups that received treatments compared with the control group. At the same time, there are significant count changes between treated groups illustrated in (Fig. 8A). The mean count of Sertoli cells (SC) highly significant (*p* ≤ 0.05) decreased in Group II and IV treated animals compared with the control animals, however, the SC count was slightly decreased significantly (*p* ≤ 0.05) in Group III and V compared with the control group (Fig. 8B).

### Spermatogenic index (SI)

Regarding the mean spermatogenic index (SI) there was a significant (*p* ≤ 0.05) decrease in SI of all treated groups compared with the control group, in addition, there were significant (*p* ≤ 0.05) SI changes between the treated groups in compared to each other (Fig. 8C).

### Immunohistochemical study

**Fig. 9:**
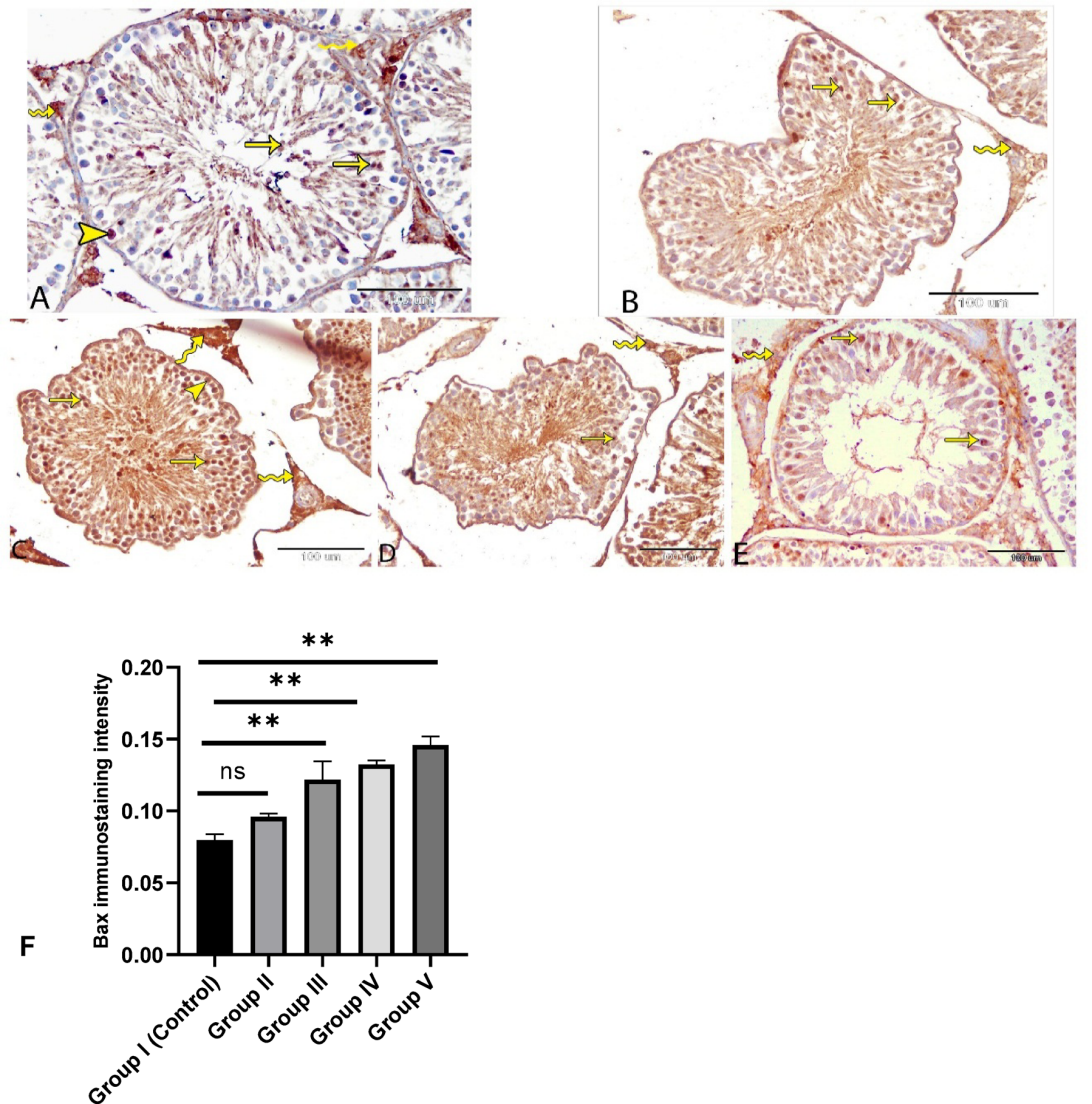
Immunohistochemical localization of Bax protein in testes of rats in control group **(A)**, group II **(B)**, group III **(C)**, group IV **(D)** and group V **(E)**. Low to moderate Bax staining was observed in germ cells (arrows) and Leydig cells (wavy arrows) in the control group **(A)**. Strong staining was observed in spermatogonia (arrow heads), spermatocytes (arrows) and Leydig cells (wavy arrows) in the experimental group **(B-E)**. **F**: Staining quantification of Bax using image j software. The treated groups are compared to control using Tukey test (*n* = 3). *P ≤ 0.05.

In the control group, Bax immunostaining appeared weak to moderate (Fig. 9A), whereas a marked increase in Bax expression was observed across all experimental groups (Fig. 9B–E). In both control and treated groups, Bax was localized to the cytoplasm and nucleus of various testicular cells, including spermatogonia, spermatocytes, differentiating spermatids, late spermatocytes, and Leydig cells, indicating its broad involvement in germ cell regulation.

**Fig. 10:**
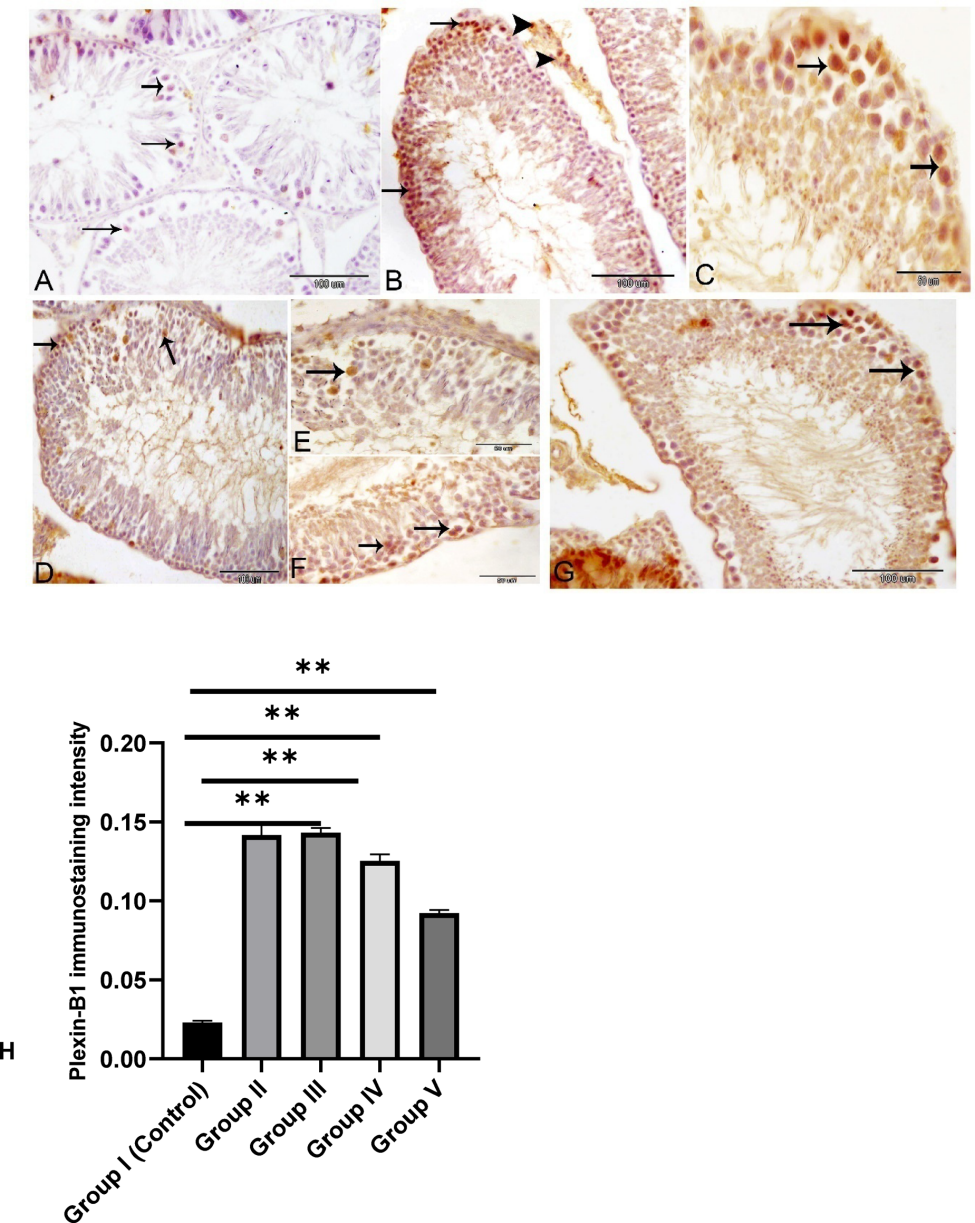
Immunohistochemical localization of Plexin-B1 protein in testes of rats in the control group **(A)**, group II **(B)**, group III **(C)**, group IV **(D)**, and group V **(E)**. Plexin-B1 immunostaining is weak in a few spermatogenic cells (arrows) in the control group **(A)**. Strong staining is observed in spermatogonia, spermatocytes (arrows), and Leydig cells (arrow heads) in the experimental group **(B-E)**. **H**: Staining quantification of Plexin-B1 using image j software. The treated groups are compared to control using Tukey test (*n* = 3). *P ≤ 0.05.

Plexin-B1 immunostaining, by contrast, was faint and limited to a few spermatogenic cells in the control group. However, in the experimental groups, Plexin-B1 expression expanded significantly, appearing in multiple cell types such as Sertoli cells, Leydig cells, and various spermatogenic cells. In Sertoli cells, staining was evident in both the cytoplasm and the cell membrane, while in Leydig cells, it was confined to the cytoplasm. Notably, Plexin-B1 was also detected in spermatogonial stem cells, suggesting a potential role in guiding germ cell migration and differentiation during spermatogenesis. (Fig. 10).

## Discussion

Lactic acid bacteria (LAB) are crucial in the fermentation and pickling processes of white soft cheeses such as Domiati, Feta, and other brined varieties. They contribute to preservation, flavor development, texture modification, and safety. LAB hydrolyzes milk proteins (caseins) into peptides and free amino acids, which enhance the cheese’s flavor and texture^28^.

The decrease in total solids of cheese during storage may be related to the conversion of some lactose (a solid) to lactic acid (a liquid) by the bio-metabolism of lactic acid bacteria. Moreover, due to the degradation of protein and fat with the progression of storage time^32^. Our results agree with the previous studies reported by Abdalla and Mohamed^33^ who reported that the total solids content of cheese decreased from 48.47% to 47.20%.

In our results, the protein/DM values increased with the progress of storage time and as well as the addition of lactic acid strains (e, g., incubated with *L. helveticus*,* L. rhamnosus*, and *S. thermophilus S*_*3855*_). The same trend was observed by Moneeb et al^34^. who reported the mean of protein content increased from 17.59% to 19.27%, and reverse results were reported by Effat et al^35^.. The addition of bacterial strains to cheese led to an increase in protein content^36^, whereas the cultured cheeses had higher protein/DM content when compared with control cheese.

A progressive decline in total solids during storage was associated with a decrease in fat levels. These results corresponded with those found by Hayalogluet al^32^. who observed that the SF cheeses had a little lower fat-in-DM content than the other cheeses and who attribute that to the increased moisture content. These results were less than those obtained by Dhuol and Hamid^37^.

Rats fed on white soft cheese cultured by different strains of LAB ranked noticeable decreases in serum TC content. Similar results were obtained by Al-Awwad et al^38^.. The TC-lowering effect of lactic acid bacteria is due to the inhibition of 3-hydroxy-3-methyl glutamyl CoA reductase, which is a rate-limiting enzyme responsible for endogenous cholesterol biosynthesis in the body^39^.

The data showed a clear reduction in triglyceride (TG) levels across the different rat groups, consistent with findings by Beena and Prasad^40^. This reduction may be attributed to increased bile acid production from cholesterol, which lowers circulating lipid levels, including triglycerides, and reduces their reabsorption. Consequently, the liver compensates by increasing the activity of bile salt hydrolase (BSH), leading to greater bile acid excretion. LAB, known for their ability to hydrolyze bile salts^41^. Several studies have reported that rats fed cultured milk products exhibit significantly lower triglyceride concentrations of^42,43^.

The significant reduction in serum triglyceride (TG) levels observed—particularly in Group V (fed on *S. thermophilus* S3855 cheese), from 78.26 to 55.57 mg/dL—may contribute to the reproductive changes seen in this study. Lipids, including triglycerides and cholesterol, play central roles in testicular steroidogenesis and in maintaining the integrity of germ cell membranes. In rat models, dietary lipid composition has been shown to influence both the activity of key steroidogenic enzymes and testosterone output: for example, feeding different sources of dietary oil significantly altered testicular neutral and polar lipid contents, raised testosterone, and increased 3β- and 17β-hydroxysteroid dehydrogenases when compared to less favorable fat sources^44^.

Disruption of lipid metabolism whether by excess or deficiency, can impair germ cell development. High-fat diets in rats lead to metabolic disturbances in Sertoli cells, including impaired glucose and lipid metabolism, and correlate with reduced sperm concentrations and impaired spermatogenesis^45^. Moreover, altered serum TG (and other lipids) have been associated with changes in sperm motility, density, and hormonal regulatory pathways in both animal models and human studies^46^.

In the present study, while TG levels were reduced rather than elevated (which is often the case in dyslipidemia models), this decrease might reflect altered lipid availability necessary for membrane synthesis, steroid hormone production, or general germ cell nourishment. It is possible that excessively low triglyceride levels or disruption of lipid transport or local lipid metabolism could reduce substrate availability for Leydig cell steroidogenesis, compromise Sertoli cell support functions, or affect germ cell membrane composition, thereby magnifying apoptotic responses and contributing to the histopathological degenerations observed.

Therefore, the TG reduction in Group V (and perhaps the other treated groups) should not be dismissed as merely a metabolic side effect but considered as a potential mediator of reproductive impairment. In future work, measurement of lipid profiles within testicular tissue, the status of steroid hormones, and direct sperm functional parameters would help clarify the mechanism by which changes in systemic TG levels map to local testicular effects.

Previous studies have reported some hazards associated with the use of probiotics. There have been reports of Lactobacillus bacteremia and sepsis related to probiotic combinations, Bacillus subtilis, or *Lactobacillus rhamnosus GG.*^47,48^. Moreover, Streptococcus and Lactobacillus probiotics induced endocarditis^47^.

Significant concerns regarding the safety of probiotics were brought up by a clinical investigation^49^. There was a significant mortality incidence among the trial participants in the probiotic group, which was linked to intestinal ischemia. Even though the blood flow was already minimal, the scientists postulated that the administration of probiotic bacteria increased the oxygen consumption in the gut mucosa. Conversely, probiotics cause an inflammatory response in the small intestine, which lowers capillary blood flow capacity.

Concerning the histopathological changes of the testis, the histological pattern of the testis of the control group showed the normal histological structure of seminiferous tubules (ST) including the shape and arrangement of their cellular components. Spermatogonia (Sg) and Sertoli cells (SCs) rest on intact basement membranes. Large primary spermatocytes (PS) with characteristic large rounded nuclei, round spermatids, elongated spermatids, and late-stage sperms (S) attached to the apices of SCs were seen^12^.

The testis is the organ of fertility and any alteration in its structure of course reflected on function. Our results showed multiple systemic alterations in testes which alter their morphology in experimental groups, in addition to several vascular and inflammatory changes. Our findings agreed with previous studies, which found many side effects after probiotic administration^50,51^. The testes of rats that were fed with Domiati cheese stored at room temperature showed atrophy of tissues and disturbance of the layers of spermatogenic cells^12^.

Our previous publication clarified several histopathological alterations in the liver and kidneys of rats fed with cultured white soft cheese in all experimentally treated groups away from control rats^7^.

Regarding immunohistochemical observation, the BAX gene, known as Bcl-2 Associated X-protein, is a pro-apoptotic protein and is a member of the Bcl-2 gene family that regulates the intrinsic pathway of apoptosis^52^. In the present study, moderate Bax immunoreactivity was observed in all germ cells in the control group. However, strong Bax immunoreactive staining was detected in the other experimental groups. Our results demonstrated the presence of bacteria in the cheese induced apoptosis in the exposed experimental groups. Apoptosis (programmed cell death) is a natural process that happens in response to different stimuli, including infection and cellular stress. Bax plays a key role in initiating apoptosis by promoting the release of cytochrome from mitochondria, which then activates caspases and leads to cell death^53^. It’s believed that the presence of bacteria in the cheese triggered an immunological response in the exposed group(s), resulting in elevated Bax expression and subsequent apoptosis.

Plexins are a family of transmembrane receptors that bind to and are activated by members of the Semaphorin family of proteins. Semaphorins are a large family of secreted and membrane-bound proteins that are involved in a wide range of biological processes^54^. Nine plexins have been identified in vertebrates. These plexins are categorized into four subfamilies based on their structural characteristics, namely Plexin-A to Plexin-D^55^. Plexins studied are widely expressed differently in neuronal and non-neuronal tissues. In the testis, Plexin-B1 is expressed in the developing sex cords and is supposed to play a role in regulating germ cell migration and differentiation^56^. Plexin-B1 in developing sex cords during testis development suggests that it may play an important role in the proper formation and function of the testis. Our results demonstrated that the expression of Plexin-B1 was extensive in all experimental groups except the control. The overexpression of the Plexin-B1 gene contributes to prostate cancer progression^57^ through activation of the oncogenes c-Met and ErbB2 [49]. These findings lead us to conclude that the upregulation of Plexin-B1 in the experimental groups may be associated with pathways involved in abnormal cellular signaling and potential reproductive dysfunction.

## Conclusions

This study emphasizes the benefits and possible hazards of probiotic bacteria, especially when they are administered through white soft cheese. While probiotic strains such as *Lactobacillus helveticus*,* L. rhamnosus*,* and Streptococcus thermophilus* are known for their roles in improving gut health and modulating lipid profiles, our findings suggest possible risks to male reproductive health, as evidenced by histopathological and immunohistochemical alterations in testicular tissue. Increased expression of pro-apoptotic markers like Bax and the unusual expression of Plexin-B1 indicate mechanisms involving apoptosis and disrupted germ cell function. Moreover, the current study emphasizes the need for precaution, as probiotic use has been correlated with systemic infections. Therefore, while probiotics offer promising health benefits, their safety profiles must be carefully evaluated.

## Data Availability

The datasets used and/or analyzed during the current study are available from the corresponding author on reasonable request.

## References

[1] Massouras, T., Zoidou, E., Baradaki, Z. & Karela, M. Physicochemical, Microbiological and sensory characteristics of white Brined cheese ripened and preserved in large-capacity stainless steel tanks. *Foods* **12**, 2332 (2023). 37372543 10.3390/foods12122332 PMC10296873

[2] McSweeney, P. L. H. *Cheese problems solved* (Elsevier, 2007).

[3] Coronado, K. A. G., García-Torres, S. M., Caldas-Cueva, J. P., Campos-Montiel, R. G. & Ludeña-Urquizo, F. E. Physicochemical, textural, and sensory characteristics of Peruvian fresh cheese with added probiotic lactic acid bacteria. (2023).

[4] de Andrade, D. P. et al. Microencapsulation of presumptive probiotic bacteria Lactiplantibacillus plantarum CCMA 0359: technology and potential application in cream cheese. *Int. Dairy J.* **143**, 105669 (2023).

[5] Terzić-Vidojević, A. et al. Diversity of non-starter lactic acid bacteria in autochthonous dairy products from Western Balkan Countries-technological and probiotic properties. *Food Res. Int.* **136**, 109494 (2020). 32846575 10.1016/j.foodres.2020.109494

[6] Vaarala, O. REVIEW immunological effects of probiotics with special reference to lactobacilli. *Clin. Exp. Allergy* **33**(12), 1634–40 (2003). 10.1111/j.1365-2222.2003.01835.x 14656348

[7] Salman, K. H., Ali, F. A. Z. & Elhanbaly, R. Effect of cultured white soft cheese on the histopathological changes in the kidneys and liver of albino rats. *Sci. Rep.* **12**, 2564 (2022). 35169197 10.1038/s41598-022-06522-y PMC8847355

[8] Sharma, R. & Agarwal, A. Spermatogenesis: an overview. *Sperm Chromatin: Biol. Clin. Appl. Male Infertility Assist. Reproduction* **33**(12), 1634 (2011).

[9] Ruthig, V. A. & Lamb, D. J. Updates in Sertoli cell-mediated signaling during spermatogenesis and advances in restoring Sertoli cell function. *Front. Endocrinol.* **13**, 897196 (2022). 10.3389/fendo.2022.897196 PMC9114725 35600584

[10] Inoue, M., Baba, T. & Morohashi, K. -i. Recent progress in Understanding the mechanisms of Leydig cell differentiation. *Mol. Cell. Endocrinol.* **468**, 39–46 (2018). 29309805 10.1016/j.mce.2017.12.013

[11] Ge, R., Chen, G. & Hardy, M. P. The role of the Leydig cell in spermatogenic function. *Molecular Mech. Spermatogenesis* **636**, 255–269 (2008). 10.1007/978-0-387-09597-4_14 19856172

[12] Cao, X. et al. Urban fine particulate matter exposure causes male reproductive injury through destroying blood-testis barrier (BTB) integrity. Toxicol. Lett.**266**, 1–12 (2017). 10.1016/j.toxlet.2016.12.004 27939690

[13] Milk, I. D. F. Cream and Evaporated Milk-Total Solids; Standard 21 B; Int. *Dairy Fed.: Brussels, Belgium* (1987).

[14] Rațu, R. N. et al. Study on the chemical composition and nitrogen fraction of milk from different animal species. *Scientific Papers Ser. D Anim. Science* **64**, 374–379 (2021).

[15] Association of Official Analytical, C. Official methods of analysis of the Association of Official Analytical Chemists. Vol. 11. The Association, (2000).

[16] Parasuraman, S., Raveendran, R. & Kesavan, R. Blood sample collection in small laboratory animals. *J. Pharmacol. Pharmacotherapeutics*. **1**, 87 (2010). 10.4103/0976-500X.72350 PMC3043327 21350616

[17] Ng, V. & Effects of Disease on Clinical Laboratory Tests., Vol. 1 and 2. DS Young and RB Friedman, eds. Washington, DC: AACC Press, 199.00(159.00 AACC members), softcover. ISBN 1-890883-45-X. *Clinical Chemistry* **48**, 682–683 (2002). (2001).

[18] Fossati, P. & Prencipe, L. Serum triglycerides determined colorimetrically with an enzyme that produces hydrogen peroxide. *Clin. Chem.* **28**, 2077–2080 (1982). 6812986

[19] Sayed, R. K. A. et al. Lack of NLRP3 inflammasome activation reduces age-dependent sarcopenia and mitochondrial dysfunction, favoring the prophylactic effect of melatonin. *Journals Gerontology: Ser. A*. **74**, 1699–1708 (2019). 10.1093/gerona/glz079 30869745

[20] Underwood, W. & Anthony, R. AVMA guidelines for the euthanasia of animals: 2020 edition. *Retrieved March*. **2013**, 2020–2021 (2020).

[21] Carleton, H. M., Drury, R. A. B. & Wallington, E. A. Carleton’s histological technique. *(No Title)* (1967).

[22] Soliman, H. M., Wagih, H. M., Attia, G. M. & Algaidi, S. A. Light and electron microscopic study on the effect of antischizophrenic drugs on the structure of seminiferous tubules of adult male albino rats. *Folia Histochem. Cytobiol.* **52**, 335–349 (2014). 25535927 10.5603/FHC.a2014.0038

[23] Ibrahim, I. A., Hussein, M. M., Hamdy, A. & Abdel-Maksoud, F. M. Comparative morphological features of Syrinx in male domestic fowl Gallus Gallus domesticus and male domestic pigeon Columba Livia domestica: A Histochemical, Ultrastructural, scanning electron microscopic and morphometrical study. *Microsc Microanal*. **26**, 326–347. 10.1017/S1431927620000021 (2020). 32000880 10.1017/S1431927620000021

[24] Johnsen, S. G. Testicular biopsy score count–a method for registration of spermatogenesis in human testes: normal values and results in 335 hypogonadal males. *Hormone Res. Paediatrics*. **1**, 2–25 (1970). 10.1159/000178170 5527187

[25] Oliveira Filho, A. B., Souza, R. S., Azeredo-Oliveira, M. T. V., Peruquetti, R. L. & Cedenho, A. P. Microdissection testicular sperm extraction causes spermatogenic alterations in the contralateral testis. (2010). 10.4238/vol9-3gmr860 20662155

[26] Filipiak, E. et al. Estrogen receptor alpha localization in the testes of men with normal spermatogenesis. *Folia Histochem. Cytobiol.* **50**, 340–345 (2012). 10.5603/19743 23042285

[27] Gibson-Corley, K. N., Olivier, A. K. & Meyerholz, D. K. Principles for valid histopathologic scoring in research. *Vet. Pathol.* **50**, 1007–1015 (2013). 23558974 10.1177/0300985813485099 PMC3795863

[28] El-Baradei, G., Delacroix-Buchet, A. & Ogier, J. C. Biodiversity of bacterial ecosystems in traditional Egyptian domiati cheese. *Appl. Environ. Microbiol.* **73**, 1248–1255 (2007). 17189434 10.1128/AEM.01667-06 PMC1828670

[29] de Bringel, S. Endocrine and testicular changes induced by olanzapine in adult Wistar rats. *J. Appl. Toxicol.* **33**, 24–31 (2013). 21780154 10.1002/jat.1702

[30] Ibrahim, D. & Abdel-Maksoud, F. M. Immunohistochemical and ultrastructural features of the seasonal changes in the epididymal epithelium of camel (Camelus dromedarius). *Microsc Microanal*. **25**, 1273–1282. 10.1017/S1431927619014843 (2019). 31547896 10.1017/S1431927619014843

[31] Ibrahim, D. et al. Immunohistochemical studies for the neuronal elements in the vomeronasal organ of the one-humped camel. *J. Vet. Med. Sci.* **77**, 241–245. 10.1292/jvms.14-0424 (2015). 25319516 10.1292/jvms.14-0424 PMC4363031

[32] Hayaloglu, A. A., Guven, M., Fox, P. F. & McSweeney, P. L. H. Influence of starters on chemical, biochemical, and sensory changes in Turkish white-brined cheese during ripening. *J. Dairy Sci.* **88**, 3460–3474 (2005). 16162519 10.3168/jds.S0022-0302(05)73030-7

[33] Abdalla, M. O. M. & Mohamed, S. N. Effect of storage period on chemical composition and sensory characteristics of vacuum packaged white soft cheese. *Pakistan J. Nutr.* **8**, 145–147 (2009).

[34] Moneeb, A. H. M., Ali, A. K., Ahmed, M. E. & Elderwy, Y. Characteristics of low-fat white soft cheese made with different ratios of bifidobacterium bifidum. *Assiut J. Agricultural Sci.* **53**, 31–44 (2022).

[35] Effat, B. A. M., Mabrouk, A. M. M., Sadek, Z. I., Hussein, G. A. M. & Magdoub, M. N. I. Production of novel functional white soft cheese. *J. Microbiol. Biotechnol. Food Sci.* **1**, 1259–1278 (2012).

[36] Dafalla, A., Abdel Razig, K., Elrofaei, N. A. J. A. S. & R. J. E. T. S. Effect of types of probiotic bacteria on physiochemical properties of Sudanese white soft cheese. **78**, 83–97 (2021).

[37] Dhuol, K. R. R. & Hamid, O. I. A. Physicochemical and sensory characteristics of white soft cheese made from different levels of cassava powder (Manihot esculenta). *Int. J. Curr. Res. Acad. Rev.* **1**, 1–12 (2013).

[38] Al-Awwad, N. J., Takruri, H. R. & Yamani, M. I. Effect of probiotic hummus on blood lipids of rats. *Jordan J. Biol. Sci.* **7**, 261–267 (2014).

[39] Sudha, M. R., Chauhan, P., Dixit, K., Babu, S. & Jamil, K. Probiotics as complementary therapy for hypercholesterolemia. *Biol. Med.* **1**, 1–13 (2009).

[40] Beena, A. & Prasad, V. Effect of yogurt and bifidus yogurt fortified with skim milk powder, condensed Whey and lactose-hydrolysed condensed Whey on serum cholesterol and triacylglycerol levels in rats. *J. Dairy Res.* **64**, 453–457 (1997). 9275259 10.1017/s0022029997002252

[41] Begley, M., Hill, C. & Gahan, C. G. M. Bile salt hydrolase activity in probiotics. *Appl. Environ. Microbiol.* **72**, 1729–1738 (2006). 16517616 10.1128/AEM.72.3.1729-1738.2006 PMC1393245

[42] Hu Xu, H. X., Wang Tao, W. T., Li Wei, L. W., Jin Feng, J. F. & Wang Li, W. L. Effects of NS lactobacillus strains on lipid metabolism of rats fed a high-cholesterol diet. (2013). 10.1186/1476-511X-12-67 PMC3667092 23656797

[43] Kim BoBae, K. B. et al. Protective effects of Lactobacillus rhamnosus GG against dyslipidemia in high-fat diet-induced obese mice. (2016). 10.1016/j.bbrc.2016.03.107 27018382

[44] de Hurtado, G. E., de Alaniz, M. J. & Marra, C. A. Influence of commercial dietary oils on lipid composition and testosterone production in interstitial cells isolated from rat testis. *Lipids* **44**, 345–357. 10.1007/s11745-008-3277-z (2009). 19130109 10.1007/s11745-008-3277-z

[45] Luo, D. et al. High fat diet impairs spermatogenesis by regulating glucose and lipid metabolism in Sertoli cells. *Life Sci.* **257**, 118028. 10.1016/j.lfs.2020.118028 (2020). 32615185 10.1016/j.lfs.2020.118028

[46] Kim, N. et al. Effect of lipid metabolism on male fertility. *Biochem. Biophys. Res. Commun.* **485**, 686–692. 10.1016/j.bbrc.2017.02.103 (2017). 28235483 10.1016/j.bbrc.2017.02.103

[47] Mackay, A. D., Taylor, M. B., Kibbler, C. C. & Hamilton-Miller, J. M. T. Lactobacillus endocarditis caused by a probiotic organism. *Clin. Microbiol. Infect.* **5**, 290–292 (1999). 11856270 10.1111/j.1469-0691.1999.tb00144.x

[48] Zein, E. F. *et al.* 2 edn 195–198.

[49] Besselink, M. G. H. et al. Probiotic prophylaxis in predicted severe acute pancreatitis: a randomised, double-blind, placebo-controlled trial. *Lancet* **371**, 651–659 (2008). 18279948 10.1016/S0140-6736(08)60207-X

[50] Kunz, A. N., Noel, J. M. & Fairchok, M. P. Two cases of Lactobacillus bacteremia during probiotic treatment of short gut syndrome. *J. Pediatr. Gastroenterol. Nutr.* **38**, 457–458 (2004). 15085028 10.1097/00005176-200404000-00017

[51] Land, M. H. et al. Lactobacillus sepsis associated with probiotic therapy. *Pediatrics* **115**, 178–181 (2005). 15629999 10.1542/peds.2004-2137

[52] Apte, S. S., Mattei, M. G. & Olsen, B. R. Mapping of the human BAX gene to chromosome 19q13. 3–q13. 4 and isolation of a novel alternatively spliced transcript, BAXδ. *Genomics* **26**, 592–594 (1995). 7607685 10.1016/0888-7543(95)80180-t

[53] Renault, T. T., Floros, K. V. & Chipuk, J. E. BAK/BAX activation and cytochrome c release assays using isolated mitochondria. *Methods* **61**, 146–155 (2013). 23567751 10.1016/j.ymeth.2013.03.030 PMC3686896

[54] Takahashi, T. et al. Plexin-neuropilin-1 complexes form functional semaphorin-3A receptors. *Cell* **99**, 59–69 (1999). 10520994 10.1016/s0092-8674(00)80062-8

[55] Maestrini, E. et al. A family of transmembrane proteins with homology to the MET-hepatocyte growth factor receptor. *Proc. Natl. Acad. Sci.* **93**, 674–678 (1996). 8570614 10.1073/pnas.93.2.674 PMC40111

[56] Perälä, N. M., Immonen, T. & Sariola, H. The expression of plexins during mouse embryogenesis. *Gene Expr. Patterns*. **5**, 355–362 (2005). 15661641 10.1016/j.modgep.2004.10.001

[57] Wong, O. G. W. et al. Plexin-B1 mutations in prostate cancer. *Proc. Natl. Acad. Sci.* **104**, 19040–19045 (2007). 18024597 10.1073/pnas.0702544104 PMC2141904

